# Management of Furcal Perforation/Involvement in a Primary Molar With Irreversible Pulpitis Using Tampon Pulpotomy: A 18‐Month Healing Case Report

**DOI:** 10.1002/ccr3.72004

**Published:** 2026-02-15

**Authors:** Saeed Asgary, Fatemeh Shekarchi

**Affiliations:** ^1^ Iranian Centre for Endodontic Research, Research Institute of Dental Sciences Shahid Beheshti University of Medical Sciences, Evin Tehran Iran; ^2^ Department of Pediatric Dentistry, School of Dentistry Shahid Beheshti University of Medical Sciences Tehran Iran

**Keywords:** CEM cement, pediatric endodontics, primary molars, regenerative endodontics, tampon pulpotomy, vital pulp therapy

## Abstract

Successful management of furcal perforation in a primary molar with irreversible pulpitis can be achieved using tampon pulpotomy with calcium‐enriched mixture cement. This conservative, biologically based technique promotes long‐term tooth retention and radiographic healing, offering an alternative to extraction in complex pediatric cases.

## 1 Introduction

Maintaining the functionality and integrity of primary molars until their natural exfoliation is a fundamental objective in pediatric dentistry. These teeth serve critical roles in mastication, speech development, and, importantly, the guidance of permanent successors [1]. Historically, when primary molars develop furcal pathology, especially in the context of pulpal floor perforation and chronic inflammation, the clinical decision often tilts toward extraction. The presence of furcal radiolucency and lamina dura loss in primary teeth has long been interpreted as radiographic indicators of irreversible pulpitis or even pulp necrosis, especially when associated with a history of localized abscess or swelling [2]. Extracting such teeth typically requires subsequent space maintenance to prevent mesial drift, tipping of adjacent teeth, and subsequent malocclusion. However, extraction comes with its complications: psychological distress, financial cost, the need for additional clinical appointments, and the potential long‐term disruption of dental arch form and function [2, 3].

Contemporary developments in vital pulp therapy (VPT) have challenged these conventional management strategies by introducing biologically driven, minimally invasive alternatives. Among these, tampon pulpotomy, a technique involving the placement of a bioactive cement over persistently bleeding pulp stumps, has gained attention for its clinical feasibility and potential to preserve pulp vitality in challenging conditions [4, 5]. Unlike traditional pulpotomy, which requires cessation of bleeding within a limited timeframe to indicate healthy radicular pulp, tampon pulpotomy reframes persistent bleeding as a manageable therapeutic challenge rather than a treatment contraindication. This method leverages bioactive cements not only for their physical sealing properties but also for their biologically regenerative potential [5].

Materials such as mineral trioxide aggregate (MTA), Biodentine, and calcium‐enriched mixture (CEM) cement have revolutionized the way clinicians address inflamed or partially necrotic pulps in pediatric dentistry [6]. CEM cement, in particular, has demonstrated excellent biocompatibility, antimicrobial properties, and the ability to induce hard tissue formation, including dentin bridges and periodontal healing [7]. Its alkaline pH contributes to its antibacterial activity, while its ionic exchange capacity stimulates tissue regeneration and angiogenesis. These properties make CEM an attractive option for tampon pulpotomy, especially in cases where bleeding control is difficult or where pulpal status is ambiguous [8].

This case report describes the successful use of tampon pulpotomy with calcium‐enriched mixture (CEM) cement in a mandibular second primary molar of a 7‐year‐old boy presenting with a prior history of abscess, prolonged intraoperative bleeding indicative of irreversible pulpitis, a substantial pulpal floor perforation, and a large furcal radiolucency accompanied by lamina dura loss. Despite these findings, typically considered unfavorable prognostic indicators, conservative management was undertaken following informed parental consent.

## 2 Case History/Examination

A 7‐year‐old male patient presented to a private pediatric dental clinic with a chief complaint of intermittent pain during chewing on the lower right side. His parents reported that 5 months prior, the child had experienced facial swelling in the same region and had been treated with systemic antibiotics. However, no definitive dental treatment had followed. The patient's medical history was notable for a single episode of febrile seizures at age two, without recurrence. No current medications, allergies, or systemic diseases were reported. The child was cooperative, communicative, and generally in good health.

**FIGURE 1:**
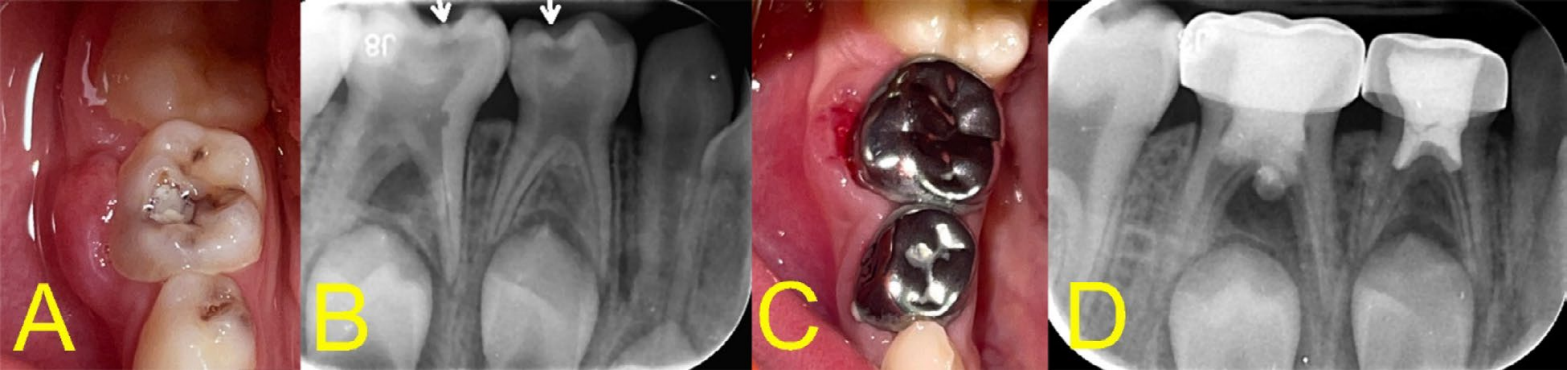
Preoperative presentation and immediate postoperative outcome. (A) Intraoral view showing buccal gingival swelling and erythema adjacent to the mandibular right second primary molar (tooth #85). (B) Preoperative periapical radiograph revealing deep occlusal caries, pulpal floor perforation, extensive furcal radiolucency, and loss of lamina dura. The developing permanent premolar appears unaffected. (C, D) Immediate postoperative photograph and radiograph after tampon pulpotomy with CEM cement and stainless‐steel crown placement. Note minor overextension of CEM cement into the furcation site.

Extraoral examination revealed no abnormalities. Intraoral clinical inspection showed localized swelling and erythema of the buccal gingiva adjacent to the mandibular right second primary molar (tooth #85) (Figure 1A). The tooth was tender to percussion and palpation, but not mobile. A large carious lesion was observed on the occlusal surface. There were no visible sinus tracts. Vital signs were within normal limits, and no systemic symptoms such as fever or lymphadenopathy were present.

The preoperative periapical radiograph revealed deep occlusal caries extending into the pulp chamber, accompanied by substantial pulpal floor perforation and a pronounced furcal radiolucency with complete loss of the lamina dura. The underlying permanent premolar exhibited normal development with no signs of involvement (Figure 1B).

## 3 Differential Diagnosis, Investigations and Treatment

Based on the clinical and radiographic presentation, a provisional diagnosis of chronic gingival abscess secondary to pulpal floor perforation and furcal pathology was established. Diagnostic ambiguity, however, was a defining feature of this case. The radiographic bone loss and previous abscess episode were suggestive of pulp necrosis, while persistent bleeding encountered during access preparation indicated retained pulp vitality, raising the possibility of irreversible pulpitis or partial necrosis.

Definitive characterization of pulpal status in this case was constrained by inherent diagnostic limitations associated with primary teeth. Cold sensibility testing was attempted but yielded no definitive response, likely reflecting both the diminished reliability of such tests in younger patients. Histological confirmation of pulpal status was likewise unavailable, given the conservative, tooth‐preserving approach adopted. As a result, the diagnosis rested on inference, integrating contradictory indicators: the presence of furcal radiolucency and a prior abscess episode suggested pulp necrosis, whereas persistent bleeding during access and the absence of a sinus tract implied at least partial pulp vitality. This diagnostic ambiguity exemplifies a central challenge in pediatric endodontics, differentiating between reversible or salvageable pulp inflammation and total necrosis in cases complicated by furcal involvement.

Two treatment options were discussed in detail with the patient's parents: (1) extraction of the affected primary molar followed by placement of a space maintainer to preserve arch integrity, and (2) conservative intervention using tampon pulpotomy with CEM cement followed by restoration with a stainless‐steel crown (SSC). The potential benefits and limitations of each approach were thoroughly explained, including the uncertain long‐term prognosis associated with VPT in cases exhibiting furcal involvement and pulpal perforation. After thoughtful consideration, the parents elected to proceed with the conservative treatment approach. Written informed consent was obtained.

Local anesthesia was administered via inferior alveolar nerve block using 2% lidocaine with 1:80,000 epinephrine. The tooth was isolated with a rubber dam, and caries excavation was performed using a sterile high‐speed bur under water spray. Upon gaining access to the pulp chamber, bleeding from the canal orifices was encountered, confirming retained pulpal vitality. The coronal pulp was gently removed with a spoon excavator, taking care to avoid disturbing the existing perforation. The chamber was irrigated with 0.5% sodium hypochlorite, followed by a sterile saline rinse.

Hemostasis was attempted using a saline‐moistened cotton pellet pressed against the canal orifices for 5 min. However, bleeding persisted. At this point, tampon pulpotomy was deemed appropriate [5]. CEM cement was prepared to a thick, putty‐like consistency and gently packed over the bleeding radicular pulp and perforation site to a depth of approximately 2 mm. A dry cotton pellet was applied with mild pressure to enhance adaptation. Notably, a minor overextension of the material into the perforation site occurred, which was left undisturbed, given the risk of displacing the cement bulk. The pulp chamber was then lined with resin‐modified glass ionomer cement, and the tooth was restored with a stainless‐steel crown cemented using a glass ionomer luting agent (Figure 1C,D). Postoperative instructions were given, and the patient was scheduled for periodic reviews.

## 4 Outcome and Follow‐Up

**FIGURE 2:**
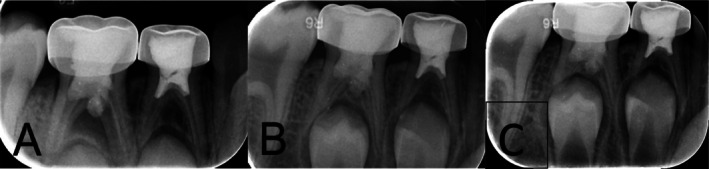
Radiographic healing progression. (A) Three‐month follow‐up radiograph demonstrating initial trabecular bone regeneration in the furcation area. (B) Twelve‐month follow‐up radiograph showing complete resolution of furcal radiolucency with trabecular bone fill, re‐establishment of lamina dura, and osseous integration of the overextended CEM cement. (C) Eighteen‐month follow‐up radiograph confirming continued trabecular bone maturation, complete lamina dura reformation, and stable integration of the extruded CEM cement without any signs of adverse tissue response.

At the 72‐h review, the child was asymptomatic, and the tooth remained functional. At the 3‐month follow‐up, intraoral examination showed complete resolution of gingival swelling and no tenderness. Radiographic assessment revealed initial trabecular bone regeneration in the furcation area (Figure 2A). At the 12‐month and 18‐month recalls, the child remained asymptomatic. The tooth was functional, with no tenderness to percussion or palpation. A periapical radiograph revealed complete trabecular bone fill in the furcal defect and reformation of a continuous lamina dura (Figure 2B,C). Importantly, the previously extruded CEM cement was embedded within the regenerated bone structure, with no evidence of adverse tissue response.

Although a CBCT scan would have provided more detailed imaging to confirm three‐dimensional bone healing, it was not performed. Given the absence of symptoms, clear evidence of healing on two‐dimensional radiographs, and the ethical considerations of exposing a child to additional radiation, the use of CBCT was deemed clinically and ethically unjustified.

## 5 Discussion

This case report shows the successful clinical and radiographic healing of a mandibular second primary molar with advanced furcal pathology, pulpal floor perforation, and a previous abscess episode following tampon pulpotomy using CEM cement. Generally, such cases with furcal radiolucency, lamina dura loss, and intraoperative bleeding are considered clear indications for extraction, mainly because of the perceived poor prognosis and risk of infection spread [9]. However, the positive outcomes observed over a 18‐month follow‐up challenge these long‐standing ideas and suggest a need to reconsider conservative biologically based VPT in complex primary molar cases.

The biological plausibility of the observed healing response is fundamentally attributed to the multifunctional properties of CEM cement. Upon hydration, its bioactive matrix releases calcium (Ca^2+^), silicate (SiO₄^4−^), and phosphate (PO₄^3−^) ions, establishing a sustained alkaline microenvironment (pH ≈ 10.5) that promotes hydroxyapatite nucleation through epitaxial crystal growth on collagen fibrils [7]. This ionic milieu concurrently upregulates expression of BMP‐2, TGF‐β1, and dentin sialophosphoprotein (DSPP) in dental pulp stem cells, driving reparative dentinogenesis and osteogenesis while generating reactive oxygen species with selective antibacterial effects against facultative anaerobes. Critically, CEM's hygroscopic expansion ensures hermetic sealing of the pulp‐perforation interface, eliminating microleakage, a primary failure mechanism in VPT. Furthermore, sustained Ca^2+^ influx suppresses pro‐inflammatory cytokines (IL‐1β, TNF‐α) and matrix metalloproteinases (MMP‐9), while simultaneously inducing VEGF‐mediated angiogenesis. This dual modulation shifts the tissue equilibrium from catabolic inflammation toward anabolic regeneration, establishing a microenvironment conducive to pulp vitality preservation and periradicular tissue repair, mechanistically explaining the radiographic evidence of trabecular bone regeneration and lamina dura reformation observed in this case [7].

The tampon pulpotomy technique is particularly useful when persistent bleeding complicates traditional pulpotomy procedures that require quick hemostasis for treatment success [4, 5, 10]. Instead of viewing ongoing bleeding as a strict contraindication, tampon pulpotomy utilizes the biological potential of the bleeding pulp tissue by placing a bioactive cement tampon that serves as both a hemostatic agent and a regenerative scaffold [4, 10]. The clot provides fibrin matrices, endogenous stem cells such as dental pulp stem cells, and growth factors, supporting reparative processes [11]. This shift in approach aligns with emerging research suggesting that irreversible pulpitis in primary teeth may be a spectrum, where inflammation with remaining viable pulp tissue can be rescued through bioactive treatments, especially if bacterial ingress is properly sealed [12]. From a clinical perspective, tampon pulpotomy offers significant advantages over extraction and space maintenance, which are associated with increased treatment sessions, higher costs, potential psychological trauma in pediatric patients, and risks of space loss or malocclusion necessitating orthodontic interventions. By preserving the primary molar, tampon pulpotomy supports occlusal function, mastication, speech development, and arch integrity, reducing the need for additional appliances and interventions [10].

An additional noteworthy finding in this case was the overextension of CEM cement into the furcation perforation. Although such extrusion is typically viewed as a procedural complication, it elicited no clinical symptoms or radiographic signs of adverse tissue response during follow‐up. While periapical radiographs suggested bony regeneration adjacent to the extruded material, the absence of three‐dimensional imaging limits definitive assessment of bone density and volume. Ethical considerations precluded CBCT acquisition in this asymptomatic pediatric patient. Furthermore, although the radiographic appearance may imply biocompatibility, the histological nature of the tissue, material interface remains unverified. Therefore, while the lack of complications supports the material's clinical tolerance, precise placement remains critical to minimize risks of foreign body reactions or unintended tissue interference.

Despite these promising outcomes, the generalizability of this single case report is limited. The 18‐month follow‐up, while suggesting healing, is not long enough to evaluate long‐term effects on succedaneous tooth eruption, potential late‐stage material degradation, or rare adverse outcomes. Additionally, reliance on two‐dimensional radiographs, although ethically justified to minimize radiation exposure in children, limits volumetric assessment of bone healing that could be better understood with CBCT.

Future research priorities include well‐designed randomized controlled trials comparing tampon pulpotomy to extraction with space maintainers, evaluating not only clinical and radiographic success but also cost‐effectiveness, patient‐reported outcomes, arch development, and quality of life. Comparative studies examining different bioactive materials (e.g., CEM, MTA, Biodentine) for mechanical properties and biological performance in perforation repair will further refine clinical protocols. Until such evidence is available, tampon pulpotomy should be viewed as a biologically plausible, ethically justifiable, and conservative treatment option for primary molars with advanced pulpal and furcal involvement. This is particularly relevant when the consequences of extraction are significant or when patients and caregivers prefer conservative care.

## 6 Conclusion

Tampon pulpotomy using CEM cement may represent a viable and ethically sound alternative to extraction for primary molars with signs of advanced pulpal and furcal pathology. This case demonstrated complete clinical/radiographic healing over 18 months, despite diagnostic ambiguity, cement overextension, and initially poor prognostic indicators. While the results are encouraging, caution must be exercised in extrapolating these findings. Further rigorous trials are essential to validate tampon pulpotomy as a mainstream treatment for furcally involved primary molars.

## Author Contributions

**Saeed Asgary:** conceptualization, investigation, methodology, project administration, supervision, writing – original draft. **Fatemeh Shekarchi:** data curation, investigation, methodology, project administration, writing – original draft, writing – review and editing.

## Funding

The authors have nothing to report.

## Disclosure

Patient Perspective: The child's parents expressed satisfaction with the outcome, noting that the procedure avoided extraction and maintained the tooth's function without complications.

## Ethics Statement

The authors have nothing to report.

## Consent

Written informed consent was obtained from the patient's parents for publication of this case report and accompanying images.

## Conflicts of Interest

The authors declare no conflicts of interest.

## Data Availability

The data supporting the findings of this case report are available from the corresponding author upon reasonable request.
